# Subtypes of Neurohypophyseal Nonapeptide Receptors and Their Functions in Rat Kidneys

**DOI:** 10.32607/actanaturae.10943

**Published:** 2020

**Authors:** A. V. Kutina, A. A. Makashov, E. V. Balbotkina, T. A. Karavashkina, Yu. V. Natochin

**Affiliations:** Sechenov Institute of Evolutionary Physiology and Biochemistry of the Russian Academy of Sciences, Saint Petersburg, 194223 Russia

**Keywords:** kidney, vasopressin, receptors, bioinformatics, ion excretion, water reabsorption

## Abstract

The nonapeptides of neurohypophysis, vasotocin and mesotocin, detected in most vertebrates, are replaced by vasopressin and oxytocin in mammals. Using bioinformatics methods, we determined the spectrum of receptor subtypes for these hormones in mammals and their physiological effects in the kidneys of rats. A search for sequences similar to the vertebrate vasotocin receptor by proteomes and transcriptomas of nine mammalian species and the rat genome revealed three subtypes of vasopressin receptors (V1a, V1b, and V2) and one type of oxytocin receptors. In the kidneys of non-anesthetized rats, which received a water load of 2 ml per 100 g of body weight, three effects of vasopressin were revealed: 1) increased reabsorption of water and sodium, 2) increased excretion of potassium ions, and 3) increased excretion of sodium ions. It has been suggested that each of the effects on the kidney is associated with selective stimulation of the vasopressin receptor subtypes V2, V1b, and V1a depending on the concentration of nonapeptide. In experiments on non-anaesthetized rats with a water load, the injection of oxytocin reduces the reabsorption of solute-free water in the kidneys and increases the excretion of sodium ions. The possible physiological mechanisms behind the realization of both effects with the participation of a single type of oxytocin receptors are being analyzed. Thus, the spectrum of activated receptor subtypes varies depending on the current concentration of neurohypophyseal hormones, as a result of which the predominant effect on renal function changes, which ensures precise regulation of water-salt homeostasis.

## INTRODUCTION

Neurohypophyseal nonapeptides affect the functions of various organs and systems in mammals and participate in the regulation of social behavior [1]. Among the peripheral effects induced by these hormones, an important role is played by the regulation of the renal function to maintain water-salt homeostasis [2]. In the neurohypophysis of most vertebrates, vasopressin-like (vasotocin, vasopressin, lysipressin, and phenipressin) and oxytocin-like (oxytocin, mesotocin, isotocin, and glumitocin) hormones are secreted [3]. Vasopressin-like peptides contain a basic amino acid residue at position 8 (Arg or Lys), and oxytocin-like peptides contain a neutral amino acid residue (Leu, Ile, or Pro) [4]. The vasopressin in mammals and humans is involved in the regulation of kidney function, which enhances the reabsorption of water, urea, and sodium [5]. The main peripheral effects of oxytocin include the uterotonic effect [6] and the stimulation of milk ejection [7]. The introduction of high doses of vasopressin and oxytocin reveals their natriuretic action [8, 9, 10], and injections Subtypes of Neurohypophyseal Nonapeptide Receptors and Their Functions in Rat Kidneys A. V. Kutina*, A. A. Makashov, E. V. Balbotkina, T. A. Karavashkina, Yu. V. Natochin Sechenov Institute of Evolutionary Physiology and Biochemistry of the Russian Academy of Sciences, Saint Petersburg, 194223 Russia *E-mail: kutina_anna@mail.ru Received February 27, 2019; in final form, February 11, 2020 DOI: 10.32607/actanaturae.10943 Copyright ® 2020 National Research University Higher School of Economics. This is an open access article distributed under the Creative Commons Attribution License,which permits unrestricted use, distribution, and reproduction in any medium, provided the original work is properly cited. of low doses of oxytocin have a hydrouretic effect [11]. We previously demonstrated that the introduction of vasotocin (the hormone of the neurohypophysis of nonmammalian vertebrates) in mammals causes an intense natriuresis, significantly exceeding that under the action of vasopressin and oxytocin [12]. Vasotocin analogs with selective antidiuretic and natriuretic effects (increase in the fractional sodium excretion from 0.5% to 15–20%) were synthesized and characterized [13]. Peptides have also been identified that increase the excretion of potassium ions by the kidneys [12]. During the evolution of vertebrates, the structure of both the neurohypophyseal hormones and their corresponding receptors changed.

It is important to understand which subtypes of receptors mediate the effects of nonapeptides and their analogs in the kidney. Are they mediated by the action of the peptides on known receptor subtypes of vasopressin (V2, V1a, and V1b) and oxytocin [14] or are there other subtypes of receptors? A new mouse receptor with a higher affinity for vasotocin than vasopressin and oxytocin was described [15]. The aim of this study was to use bioinformatics to determine the spectrum of receptor subtypes for peptides of the vasopressin and oxytocin family in mammals, as well as to identify any possibility of reproducing the effects of vasotocin analogs in rats by the administering of various doses of their natural hormones, vasopressin and oxytocin.

## EXPERIMENTAL

The homologs were searched in the rat genome (*Rattus norvegicus*) and in proteomes and transcriptomes of nine mammals: rat, human (*Homo sapiens*), chimp (*Pan troglodytes*), orangutan (*Pongo abelii*), gibbon (*Nomascus leucogenys*), dog (*Canis lupus familiaris*), mouse (*Mus musculus*), opossum (*Monodelphis domestica*), and platypus (*Ornithorhynchus anatinus*). Genomes, proteomes, and transcriptomes were selected from the NCBI’s Genome resource (http://www.ncbi.nlm.nih. gov/genome/). The list of the datasets used is shown in *Table 1*. The hidden Markov model-based nHMMER and pHMMER tools [16] and the original shell script were used to conduct a homology search. The degree of homology was evaluated using the e-value and score, which were automatically assigned by the program based on the internal algorithms. The Markov model generated based on the amino acid and nucleotide sequences of the vasotocin receptors (V_1a_ subtype) of various vertebrates was used as a query. An e-value threshold was set at the level of 1e^-3^ for amino acid alignments and 1e^-10^ for nucleotide alignments, according to published methods [17]. The sequences below the thresholds were not taken into account. All of the sequences found were ranked in decreasing order according to their similarity to the analyzed sequence (score values). The list was visualized as a chart to determine the score values threshold. The sequences above the selected threshold and two sequences below were further analyzed. All of the homologous sequences were collected in a single FASTA file. Multiple alignments of the homologs found were generated using the MAFFT alignment software package [18]. The L-INS-i algorithm was used as it was the most accurate for datasets with 200 or fewer sequences [19]. The output FASTA file was converted into the NEXUS format using the Alignment Converter web tool (http://www.ibi. vu.nl/programs/convertalignwww/) for further utilization. A Bayesian reconstruction of the phylogeny was conducted using the MrBayes software package [20] for the NEXUS file obtained during the previous step. A total of 300,000 generations were used for protein queries and 20,000 generations for nucleotide queries. The generation number was used because it was the most optimal for our dataset, according to previously conducted computational experiments. After all of the generations, the standard deviation of split frequencies fell below 0.01 (this standard deviation was selected based on published data [20]). A cladogram with the posterior probabilities for each split and a phylogram with the mean branch lengths were generated and printed in NEXUS files. The visualization and editing of the trees was performed using the FigTree tool (github. com/rambaut/figtree/). Multiple alignments of amino acid sequences of the rat vasopressin, oxytocin, and neuropeptide S receptors were conducted using Clustal Omega 1.2.4 (https://www.ebi.ac.uk/Tools/msa/ clustalo/).

**Table 1:** List of the versions of proteomes, transcriptomes, and genomes used

Species	Version
Homo sapiens	GRCh38.p12
Pan troglodytes	Clint_PTRv2
Pongo abelii	Susie_PABv2
Nomascus leucogenys	Nleu_3.0
Canis lupus familiaris	CanFam3.1
Rattus norvegicus	Rnor_6.0
Mus musculus	GRCm38.p6
Monodelphis domestica	MonDom5
Ornithorhynchus anatinus	Ornithorhynchus_anatinus-5.0.1

Physiological experiments were conducted on female Wistar rats weighing 180-230 g. There were 10 animals in each series. The rats received standard pelleted chow (Melkombinat, Russia) and water *ad libitum*. On the evening before the experiment, the rats were not fed, but they retained access to water. Housing of the animals and conducting the experiments were carried out in accordance with Russian and international rules for the use of laboratory animals. On the days of the experiments, the estrous cycle phases of the animals were determined by a microscopy of vaginal smears. Rats in proestrus, estrus, metestrus, and diestrus, on average, had ratios of 15 ± 6%, 11 ± 5%, 26 ± 6%, and 48 ± 7%, respectively. Vasopressin (Sigma-Aldrich, St. Louis, MO, USA), oxytocin (Sigma-Aldrich, USA), selective agonists of oxytocin (Carbetocin, Tocris, Bristol, UK), and V_1a_ receptors (Phe^2^, Ile^3^, Orn^8^-vasopressin, Bachem, Vista, CA, USA) at doses of 0.005, 0.015, and 0.15 nmol per 100 g of body weight (BW) were administrated intramuscularly, simultaneously with a water load (2 ml of water per 100 g BW by gavage) that was used to inhibit vasopressin secretion. Animals with a water load and intramuscular injection of saline (0.1 ml per 100 g BW) served as controls. Selective antagonists of oxytocin (Pmp^1^-Tyr(Me)^2^-Thr^4^- Orn^8^-des-Gly-NH_2_^9^-vasotocin, Bachem, USA) and V_1a_ receptors (Pmp^1^-Tyr(Me)^2^-vasopressin, Bachem, USA) were administered at a dose of 2 nmol per 100 g BW intraperitoneally, simultaneously with vasopressin (0.15 nmol/100 g BW) and the water load. The rats were placed in special individual cages to collect urine samples during spontaneous urination. The urine osmolality was determined using an Advanced Instruments 3300 microosmometer, and the concentration of sodium and potassium ions was measured on a Sherwood- 420 flame photometer. Ion excretion and water reabsorption rates were calculated over a 60-minute period after the start of the experiment. When calculating the solute-free water reabsorption, the average serum osmolality value in the rats after a water load was used, equal to 288 ± 1 mOsm/kg H_2_O. In the series of experiments, the effects of the studied hormones and the agonists of their receptor were observed in animals throughout the estrous cycle; therefore, rat renal function parameters were averaged without taking into account the cycle phase. Parameters of renal function were normalized to 100 g BW. All the data are presented as a mean ± standard error of the mean. Comparison between groups was conducted using a one-way analysis of variance followed by a t-test with Bonferroni’s correction. Differences were considered statistically significant at *p* < 0.05.

## RESULTS

**Search for vasopressin and oxytocin receptor subtypes using bioinformatics methods**

**Fig. 1:**
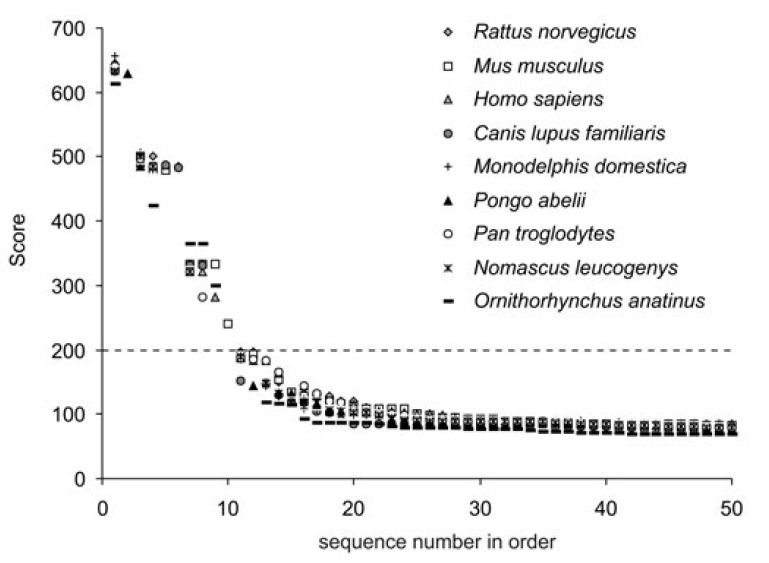
Distribution of the sequences found according to the level of similarity (score) with the average vasotocin receptor; the dotted line shows the threshold value for further analysis

Sequences found similar to the vasotocin receptor were ranked in decreasing order of the score index (*Fig. 1*). Score for sequences with the best match to the requested sequence was more than 600. Then the degree of similarity sharply decreased, and for most sequences, this index was approximately 100. Level 200 was used as the threshold value (*Fig. 1*). Sequences whose score was above the threshold were manually analyzed using the NCBI database. All of the detected proteins, which were similar in sequence to the vasotocin receptor, were annotated as various subtypes of vasopressin and oxytocin receptors. Duplicate sequences (including mutant allelic variants) were excluded from further analysis. Current versions of a series of sequences updated in the databases were found.

**Fig. 2:**
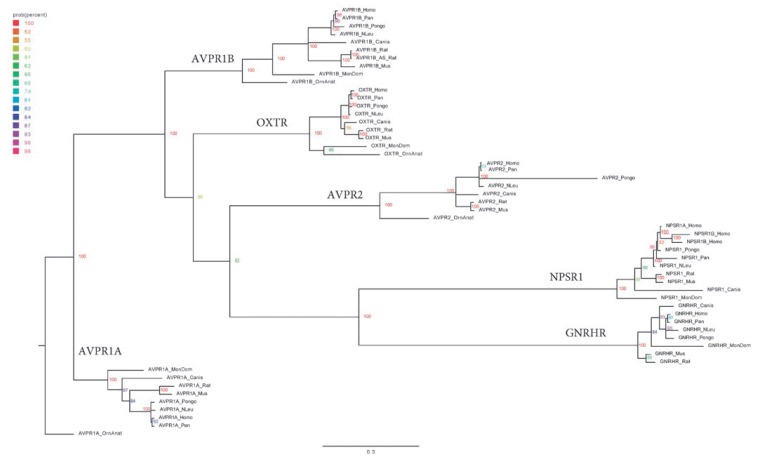
A phylogenetic tree of amino acid sequences of receptors for vasopressin, oxytocin, neuropeptide S, and the gonadotropin-releasing hormone in mammals

In all of the studied mammalian species, four different proteins were revealed among the identified sequences, which were receptors of the vasopressin and oxytocin family peptides: V_1a_ receptor (AVPR1A), V1b receptor (AVPR1B), V_2_ receptor (AVPR2), and the oxytocin receptor (OXTR). Three cases were exceptions. First, among the sequences selected at the score level, the V_2_ receptor was not found in *Pongo abelii*. A review of all of the search results yielded sequence XP_009233694.1 (score = 144.7), which is a fragment of the V_2_ receptor sequence. An updated version (XP_024096521.1) was found in the NCBI database and included in further analysis. Second, the V_2_ receptor was not found in the proteome of *Monodelphis domestica*. A manual search in the NCBI database also failed to identify this vasopressin receptor subtype in this opossum species, but a protein was found, annotated as a fragment of the V_2_ receptor in the opossum *Didelphis virginiana*. Third, in the rat (*Rattus norvegicus*), two amino acid sequences were found; NP_001276729.1 and NP_058901.3, annotated as a vasopressin receptor of the V_1b_ subtype.

**Fig. 3:**
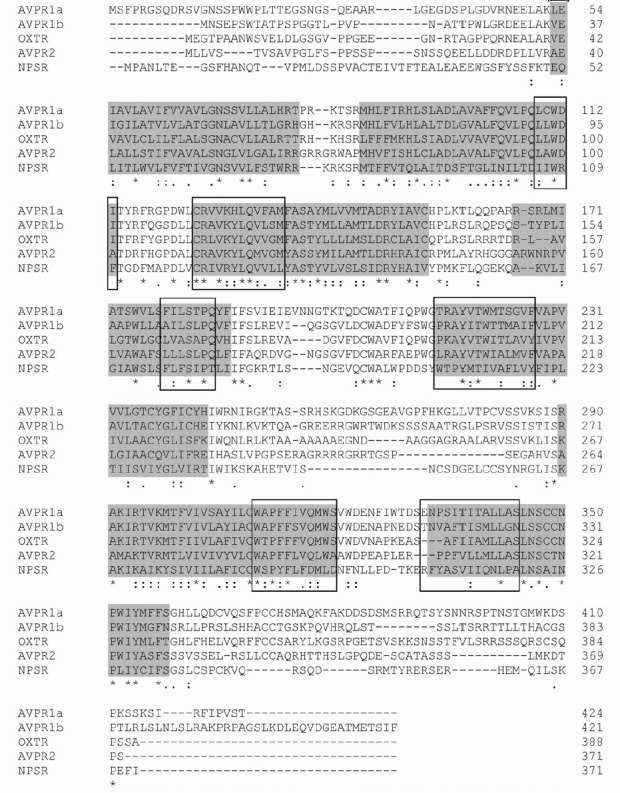
Multiple alignments of amino acid sequences of the rat vasopressin, oxytocin, and neuropeptide S receptors; 1–7 transmembrane domains are highlighted with gray shading; receptor-ligand binding sites are outlined in the frame

The closest similarity with the amino acid sequences of vasopressin family receptors in all of the studied species was established for the neuropeptide S receptor (NPSR1) and the gonadotropin-releasing hormone receptor (GNRHR). The amino acid sequences of these proteins are included in the construction of phylograms, along with all of the vasopressin and oxytocin receptor sequences found. The designations and identifiers of the amino acid sequences of the proteins used to construct the phylograms are shown in *Table 2*. When constructing the phylogram (*Fig. 2*), all of the sequences with a score above 200 were clearly divided into four clades corresponding to the V_1a_, V_1b_, and V_2_ subtypes of vasopressin receptors and the oxytocin receptor. Separate clades, sister to the V_2_ receptors, formed the receptors for neuropeptide S and the receptor for the gonadotropin-releasing hormone. The analysis of the previously published cDNA sequence of a vasotocin receptor in a mouse (GenBank: AK033957) [15] and the predicted protein structure showed that they correspond to the nucleotide and amino acid sequences of the neuropeptide S receptor. *Figure 3* shows the results of multiple alignments of the vasopressin and oxytocin receptors and the neuropeptide S receptor in the rats. The amino acid sequences of the different vasopressin and oxytocin receptors were approximately 50% identical: the V_1a_ receptor had a 54% homology with the V_1b_ receptor, a 51% with the oxytocin receptor, and a 46% with the V_2_ receptor. The neuropeptide S receptor had less similarity with vasopressin receptors (with V_1a_ = 33%, V_1b_ = 33%, and V_2_ = 30%) and oxytocin receptors (32%) and had radical substitutions at the ligand-binding sites (*Fig. 3*).

**Table 2:** Identifiers of mRNA nucleotide sequences and amino acid sequences of mammalian receptor proteins similar to the vertebrate vasotocin receptor

Species	Proteins	mRNA	Designation on the phylograms
Homo sapiens	NP_000697.1	NM_000706.4	Avpr1a_Homo
	NP_000698.1	NM_000707.4	Avpr1b_Homo
	NP_000907.2	NM_000916.3	Oxtr_Homo
	NP_000045.	NM_000054.1	Avpr2_Homo4
	NP_997055.1	NM_207172.1	Npsr1A_Homo
	NP_001287864.1	NM_001300933.1	Npsr1G_Homo
	NP_997056.1	NM_207173.1	Npsr1B_Homo
	NP_000397.1	NM_000406.2	Gnrhr_Homo
Pan troglodytes	XP_016778615.1	XM_016923126.2	Avpr1a_Pan
	XP_525039.2	XM_525039.6	Avpr1b_Pan
	XP_001144020.1	XM_001144020.5	Oxtr_Pan
	XP_001145732.2	XM_009439827.2	Avpr2_Pan
	XP_024213409.1	XM_024357641.1	Npsr1_Pan
	XP_526608.1	XM_526608.5	Gnrhr_Pan
Pongo abelii	XP_002823515.2	XM_002823469.3	Avpr1a_Pongo
	XP_002813528.1	XM_002813482.3	Oxtr_Pongo
	XP_024089895.1	XM_024234127.1	Avpr1b_Pongo
	XP_024096521.1	XM_024240753.1	Avpr2_Pongo
	XP_002818110.2	XM_002818064.3	Npsr1_Pongo
	XP_024101999.1	XM_024246231.1	Gnrhr_Pongo
Nomascus leucogenys	XP_003252777.1*	XM_003252729.3	Avpr1a_NLeu
	XP_003272998.1	XM_003272950.3	Avpr1b_NLeu
	XP_012357682.1	XM_012502228.1	Oxtr_NLeu
	XP_003279348.1	XM_003279300.2	Avpr2_NLeu
	XP_003279243.1	XM_003279195.2	Npsr1_NLeu
	XP_003268473.1	XM_003268425.1	Gnrhr_NLeu
Canis lupus familiaris	NP_001185587.1	NM_001198658.1	Avpr1a_Canis
	NP_001185588.1	NM_001198659.1	Oxtr_Canis
	XP_545695.2	XM_545695.3	Avpr1b_Canis
	NP_001003177.1	NM_001003177.1	Avpr2_Canis
	XP_022283280.1	XM_022427572.1	Npsr1_Canis
	NP_001003121.1	NM_001003121.1	Gnrhr_Canis
Rattus norvegicus	NP_444178.2	NM_053019.2	Avpr1a_Rat
	NP_058901.3	NM_017205.3	Avpr1b_Rat
	NP_001276729.1	NM_001289800.1	Avpr1b_Rat
	NP_037003.2	NM_012871.3	Oxtr_Rat
	NP_062009.1	NM_019136.1	Avpr2_Rat
	NP_001100278.1	NM_001106808.1	Npsr1_Rat
	NP_112300.2	NM_031038.3	Gnrhr_Rat
Mus musculus	NP_058543.2	NM_016847.2	Avpr1a_Mus
	NP_036054.1	NM_011924.2	Avpr1b_Mus
	NP_001074616.1	NM_001081147.1	Oxtr_Mus
	NP_062277.1	NM_019404.2	Avpr2_Mus
	NP_783609.1	NM_175678.3	Npsr1_Mus
	NP_034453.1	NM_010323.2	Gnrhr_Mus
Monodelphis domestica	XP_001372716.1	XM_001372679.3	Avpr1a_MonDom
	XP_001372263.1	XM_001372226.2	Avpr1b_MonDom
	XP_016279957.1	XM_016424471.1	Oxtr_MonDom
	XP_001365641.2	XM_001365604.4	Npsr1_MonDom
	XP_001362289.1	XM_001362252.2	Gnrhr_MonDom
Ornithorhynchus anatinus	XP_001520677.1	XM_001520627.2	Avpr1a_OrnAnat
	XP_007660695.1	XM_007662505.1	Oxtr_OrnAnat
	XP_007663815.1	XM_001520222.2	Avpr2_OrnAnat
	XP_007658276.1*	XM_007660086.1	Avpr1b_OrnAnat
	XP_016082441.1*,#	XM_016226955.1	Npsr1_OrnAnat
	NP_001116830.1	NM_001123358.1	Gnrhr_OrnAnat

Note:

^*^– incomplete sequence,

^#^– not found during automatic search, added manually from the NCBI database.

**Fig. 4:**
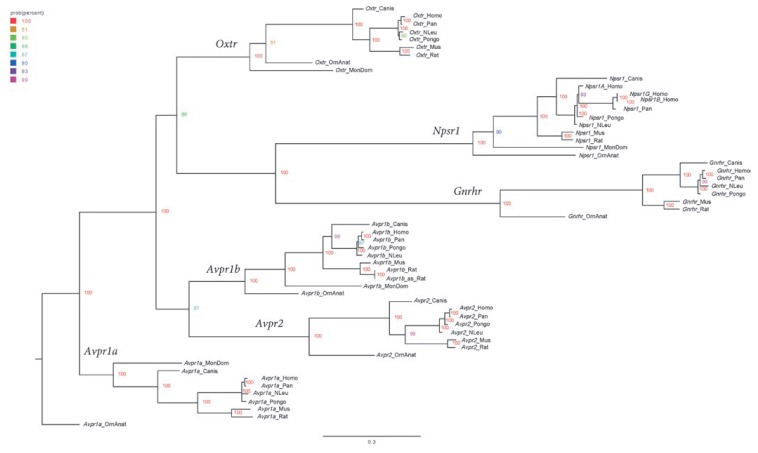
A phylogenetic tree of nucleotide sequences of mRNA of vasopressin, oxytocin, neuropeptides S, and gonadotropin- releasing hormone receptors in mammals

**Fig. 5:**
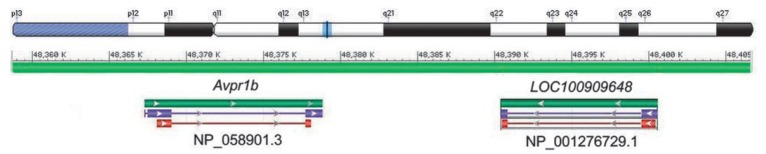
Duplication of the V_1b_ receptor gene in chromosome 13 in rat *Rattus norvegicus *(Chr 13 (NC_005112.4): 48 358 720–48 406 569)

A search for similar nucleotide sequences in the transcriptomes of nine mammalian species was conducted using the vasotocin receptor gene transcript. The mRNA detected in this way fully corresponded to the proteins detected in the previous stage in the proteome study. Two different mRNA sequences were found in *Rattus norvegicus* that encoded a V_1b_ subtype vasopressin receptor protein: NM_017205.3 and NM_001289800.1. In the proteome search, the sequence corresponding to the V_2_ receptor in the opossum (*Monodelphis domestica*) was not found. The mRNA of the gonadotropin-releasing hormone and neuropeptide S receptors were the closest to the nucleotide sequence of vasotocin receptor mRNA. The designations and identifiers of the nucleotide sequences used to construct the phylograms are shown in *Table 2*. *Figure 4* shows the mRNA phylogram of receptors of the vasopressin and oxytocin family, which is generally similar to that of proteins. When analyzing the genome in rats, five loci were identified, similar to the vasotocin receptor gene (*Table 3*). Duplication of the V_1b_ receptor gene on the long arm of chromosome 13 was detected (*Fig. 5*). The protein products from these genes are fully identical.

**Physiological effects of vasopressin and oxytocin on rat kidneys**

**Table 3:** Results of the search for genes similar to the nucleotide sequence of the vasotocin receptor in the genome of the rat (Rattus norvegicus)

Score	Chromosome (strand)	Start and end of gene (b.p.)	Transcript ID	Protein ID	Protein length (a.a.)
Avpr1a (gene ID ENSRNOG00000004400)					
852.5	7(–)	67341080-67345308	ENSRNOT 00000005829	ENSRNOP 00000005829	424
LOC100909648 (gene ID ENSRNOG00000049261)					
500.8	13(–)	48390417-48400632	ENSRNOT 00000074204	ENSRNOP 00000067252	421
Avpr1b (gene ID ENSRNOG00000048522)					
500.5	13(+)	48367307-48378831	ENSRNOT 00000074512	ENSRNOP 00000064689	421
Oxtr (gene ID ENSRNOG00000005806)					
464.3	4(–)	144403358-144416116	ENSRNOT 00000007724	ENSRNOP 00000007724	388
Avpr2 (gene ID ENSRNOG00000059862)					
238.2	X(–)	156889410-156891213	ENSRNOT 00000091495	ENSRNOP 00000071931	371

**Fig. 6:**
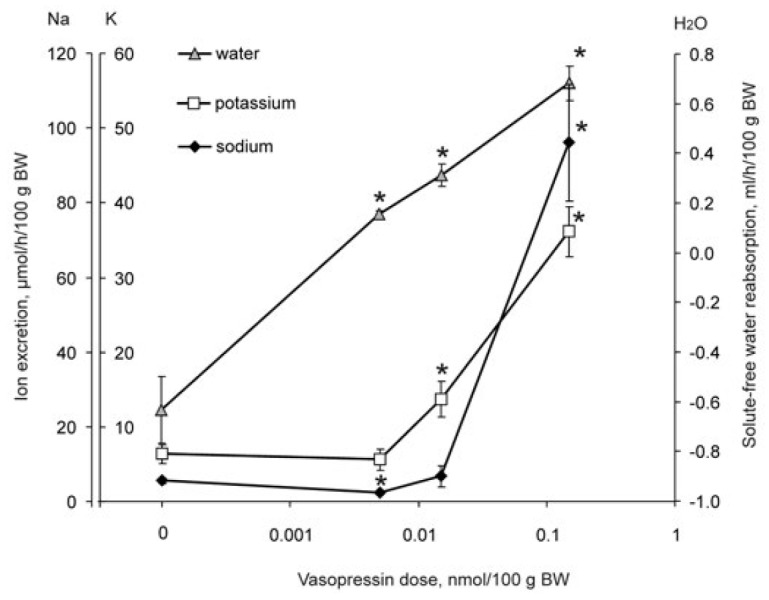
Effect of intramuscular administration of various doses of vasopressin in water-loaded rats on renal excretion of sodium, potassium ions, and reabsorption of solute-free water; * – significance of the differences (*p* < 0.05) vs control group (0 nmol/100 g BW)

The experiments on rats showed that the predominant effect of vasopressin on the excretion of sodium and potassium ions or the reabsorption of solute-free water depends on the hormone dose. This nonapeptide was administered to animals with a 2% water load, which temporarily inhibited vasopressin secretion by neurohypophysis. Injection of vasopressin at a dose of 0.005 nmol per 100 g BW had an antidiuretic effect. Despite the water load, the animals excreted concentrated urine (urine osmolality was 546 ± 31 mOsmol/kg H_2_O vs 89 ± 14 mOsmol/kg H_2_O in the group without vasopressin administration) and solute-free water reabsorption occurred in the renal tubules (*Fig. 6*). The excretion of potassium ions was the same as in the control, and the excretion of sodium ions was halved; that is, vasopressin at this dose had an antinatriuretic effect (*Fig. 6*). With a three-time increase in the vasopressin dose (up to 0.015 nmol per 100 g BW), the reabsorption of solute-free water continued to rise and the excretion of sodium did not differ from that in the control group. Under the action of the hormone at this dose, the excretion of potassium ions increased by 130% (*Fig. 6*); that is, selective kaliuresis occurred. Increasing the dose to 0.15 nmol per 100 g BW vasopressin, along with enhancing the reabsorption of solute-free water, increased the excretion of monovalent cations. The excretion of potassium and sodium ions increased, while the excretion of sodium ions prevailed (*Fig. 6*).

**Fig. 7:**
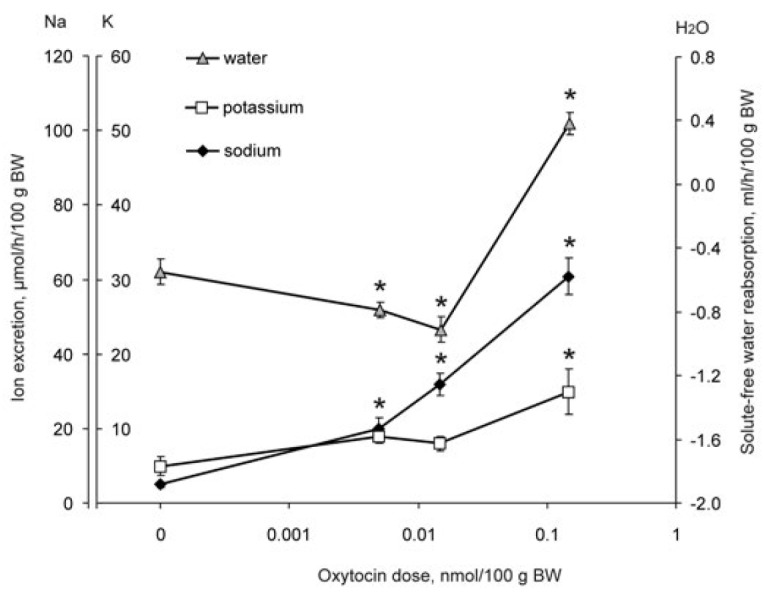
The effect of intramuscular injection of various doses of oxytocin in water-loaded rats on renal excretion of sodium, potassium ions, and the reabsorption of solute-free water; * – significance of the differences (*p* < 0.05) vs control group (0 nmol/100 g BW)

The introduction of oxytocin at doses of 0.005 and 0.015 nmol per 100 g BW under similar physiological conditions led to increased diuresis, selective naturesis, and a decrease in the reabsorption of solute-free water (*Fig. 7*). The excretion of potassium ions was similar to that in the control group. Increasing the dose of oxytocin to 0.15 nmol per 100 g BW led to an antidiuretic effect, and the reabsorption of solute-free water increased; along with an increase in the excretion of sodium ions, the excretion of potassium ions increased. The excretion of sodium ions with the action of oxytocin was lower than after the administration of vasopressin at the same dose (*Fig. 6*, *Fig. 7*).

**Fig. 8:**
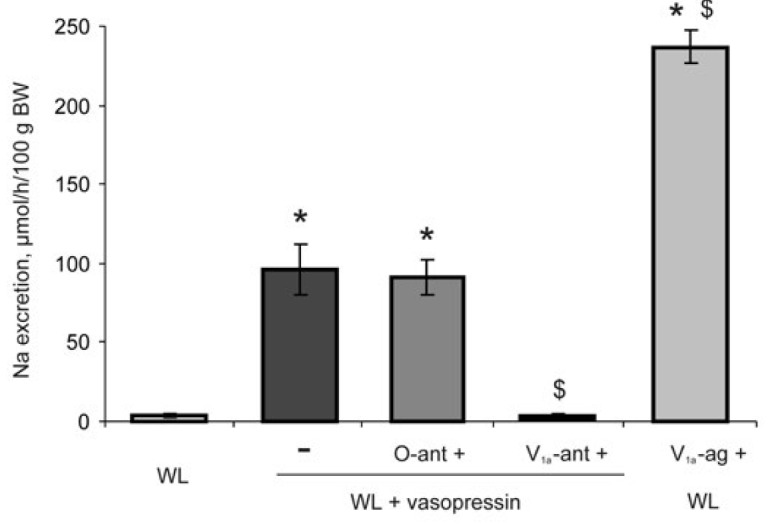
Comparison of the action of vasopressin and the V_1a_ agonist (V_1a_-ag) at a dose of 0.15 nmol per 100 g BW on sodium excretion and the influence of antagonists of V_1a_ (V_1a_-ant) and oxytocin receptors (O-ant) on the natriuretic effect of vasopressin in water-loaded rats (WL). Significant differences (*p* < 0.05): * – vs WL, $ – vs WL + vasopressin

**Fig. 9:**
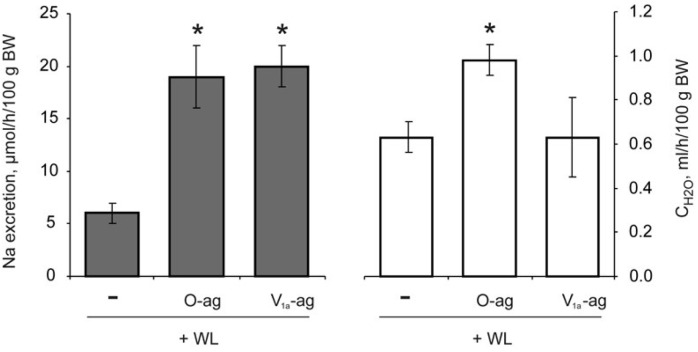
Comparison of the action of agonists of the V_1a_ receptor (V_1a_-ag) and oxytocin receptor (O-ag) at a dose of 0.015 nmol per 100 g BW in water- loaded (WL) rats on the urinary sodium excretion and clearance of solute-free water (CH_2_O). * – significance of the differences (*p* < 0.05) vs WL

The selective V_1a_ agonist reproduced the natriuretic effect of vasopressin, while sodium excretion increased significantly more than under the action of the hormone (*Fig. 8*). Blockade of V_1a_ receptors fully inhibited the development of vasopressin-induced natriuresis; the oxytocin antagonist did not exert such an effect (*Fig. 8*). Under the action of oxytocin and its receptor agonist, in contrast to the influence of the V_1a_ agonist, increased sodium excretion was accompanied by increased formation of solute-free water (*Fig. 9*).

## DISCUSSION

Various nonapeptides are synthesized in the magnocellular neurons of the hypothalamus and secreted into the blood inside the neurohypophysis: in most mammals, these are vasopressin and oxytocin; while in other vertebrates, mainly vasotocin and mesotocin [4, 21]. The effects of nonapeptides are mediated by receptors that belong to the family of membrane G-coupled receptors. According to current concepts, three consecutive duplications of receptor genes for vasopressin-like and oxytocin-like peptides have occurred in vertebrates. Based on the data obtained during the study of jawless and cartilaginous fish, it is assumed that jawed vertebrates had at least six different genes encoding receptors for the vasopressin family of hormones: five subtypes of vasopressin (vasotocin) receptors (V_1a_, V_1b_, V_2a_, V_2b_, and V_2c_) and one subtype of oxytocin receptors (in different animals, it is designated as a oxytocin, isotocin, or mesotocin receptor depending on their oxytocin-like hormone) [22]. To date, the V_2b_ receptor has been described only in fish and the V_2c_ receptor has been found in all vertebrates except mammals (it is a pseudogene in marsupials). Signal transmission at receptors of this family occurs with the participation of phospholipase C, inositol triphosphate, and calcium. The V_2a_ receptor is a notable exception that activates adenylate cyclase and stimulates the formation of cAMP as a second messenger. To answer the question as to the molecular mechanisms of polyfunctionality of vasopressin and oxytocin, a study was conducted for receptors that are similar in amino acid and nucleotide sequences to the vasotocin receptor in non-mammalian vertebrates. The data collected in the study confirmed that there exist genes in the rat genome for three V receptor subtypes (V_1a_, V_1b_, and V2 receptors) and one oxytocin receptor gene. The next closest protein in structure is the neuropeptide S receptor. Given the low degree of homology with other vasopressin and oxytocin receptors, especially the ligand- binding sites, it seems unlikely that they would be associated to the effects of vasopressin and oxytocin in the kidney, although this suggestion requires a special experimental investigation. Both the differences in the effect of increasing doses of vasopressin identified in this study (*Fig. 6*) and the different effects of vasotocin and its analogs [12] on the excretion of monovalent cations and the reabsorption of water in the kidneys of rats may be due to the different activation spectrums of the three existing V receptor subtypes. Experimental confirmation of this assumption is the result of this and previous studies using agonists and antagonists of V receptors [12]. The mechanism of the antidiuretic and antinatriuretic effects of vasopressin aimed at ensuring the osmotic concentration of urine has been the best studied. V_2_ receptors are already activated at low blood concentrations of vasopressin and increase the water permeability of collecting ducts and the activity of sodium transporters in the distal parts of the nephron [5, 23]. As the vasopressin concentration in the blood increases, it is likely that the V_1b_ and V_1a_ receptors, along with the V2 receptors, are activated and the excretion of sodium and potassium ions changes. It was previously shown that a V_1b_ receptor agonist causes an increase in potassium excretion [24], while stimulation of the V_1a_ receptor inhibits sodium reabsorption in the thick ascending limb of the loop of Henle [10, 12, 13, 25] and leads to natriuresis. This study demonstrated that the natriuretic effect of vasopressin is completely eliminated by the V_1a_ receptor antagonist. A V_1a_ agonist lacking V2 activity increases sodium excretion significantly more than vasopressin, which activates all subtypes of V receptors. The ratio of involvement of V receptor subtypes in the kidney’s physiological response to the administration of vasopressin in different doses may depend on the differences in receptor density in the membranes of the nephron tubular cells and the unequal affinities of the hormone receptors. Various methods have shown the presence of all subtypes of vasopressin and oxytocin receptors in the kidney (AVPR2 > AVPR1A > OXTR > AVPR1B) [26], and expression of the V2 receptor significantly exceeds the expression of all other receptor subtypes in this family [27]. The activation of V_1a_ receptors in rats requires a 100-fold higher concentration of vasopressin than for signaling through V2 receptors [28].

It is more challenging to discuss the mechanism of action of oxytocin in the kidney. In different doses, this nonapeptide has opposite effects on the kidney (*Fig. 7*): at a low dose, it increases the excretion of solute- free water and sodium ions, and at a higher dose, it causes an increase in the reabsorption of solute-free water and the excretion of potassium ions, with an increase in the natriuretic effect. The analysis of the proteome, transcriptome, and genome of the rat did not reveal any subtypes of the oxytocin receptor, and the mechanism of the physiological effect should be explained on the basis of the presence of one receptor subtype. According to the analysis of mRNA encoding the oxytocin receptor [27, 29], it is detected in the largest amount in the proximal nephron. A decrease in proximal tubule sodium reabsorption creates conditions for increasing solute-free water clearance [11]. This effect was described in a study of the mechanism of action of carbonic anhydrase inhibitors [30] and glucagon-like peptide-1 on the kidney [10]. Prior experiments demonstrated that oxytocin [11] causes a decrease in fluid reabsorption in the proximal tubule and that a larger volume of fluid enters the subsequent parts of the nephron. As vasopressin secretion by the neurohypophysis stops due to the water load, an oxytocin- induced increase in the volume of fluid entering the distal segment of the nephron promotes the renal excretion of solute-free water. This study showed that the enhancing effect of oxytocin on the excretion of sodium and water is reproduced with the introduction of a selective oxytocin receptor agonist. The data also indicate a difference in the mechanism of natriuresis under the action of oxytocin and vasopressin. The increase in sodium excretion is due to a decrease in sodium reabsorption in the proximal and distal parts of the nephron, respectively, when oxytocin and V_1a_ receptors are activated. The effects of the administration of high doses of oxytocin (0.15 nmol/100 g BW) are similar to those described for vasopressin and are probably associated with the effect of nonapeptide on V receptors. In contrast to oxytocin, a selective agonist of oxytocin receptors at this dose has no antidiuretic effect.

## CONCLUSION

1. An analysis of the amino acid and nucleotide sequences in proteomes and transcriptomes of nine mammalian species showed the presence of 3 subtypes of vasopressin receptors and the oxytocin receptor.

2. In a study of the rat genome using bioinformatics, genes encoding four receptor subtypes for the nonapeptides of the vasopressin and oxytocin family (*Avpr1a*, *Avpr1b*, *Avpr2*, and *Oxtr*) were found.

3. In experiments on non-anaesthetized rats that received a water load of 2 ml per 100 g BW, three effects of vasopressin in the kidney were revealed: 1) increased reabsorption of solute-free water, 2) increased excretion of potassium ions, and 3) reduced reabsorption of sodium ions. The assumption that these effects had to do with selective stimulation of the V_1a_, V_1b_, and V_2_ receptors in the kidney is substantiated.

4. In experiments on non-anaesthetized rats with water load, two effects of oxytocin in the kidney are shown: 1) reduced reabsorption of solute-free water and 2) increased excretion of sodium ions. Possible physiological mechanisms for their triggering with the participation of a single oxytocin receptor are discussed.

5. Depending on the actual concentration of hormones of the neurohypophysis, the spectrum of activated receptor subtypes and the predominant effect on renal function changes ensure precise regulation of water-salt homeostasis.
